# Dynamically characterizing individual clinical change by the steady state of disease-associated pathway

**DOI:** 10.1186/s12859-019-3271-x

**Published:** 2019-12-24

**Authors:** Shaoyan Sun, Xiangtian Yu, Fengnan Sun, Ying Tang, Juan Zhao, Tao Zeng

**Affiliations:** 1 School of Mathematics and Statistics Science, Ludong University, Yantai, 264025 China; 2 Shanghai Jiao Tong University Affiliated Sixth People’s Hospital, Shanghai, 200233 China; 3 Key Laboratory of Systems Biology, Institute of Biochemistry and Cell Biology, Chinese Academy Science, Shanghai, 200031 China; 4 Medical Laboratory, Yantaishan Hospital, Yantai, 264001 China; 5 Shanghai Research Center for Brain Science and Brain-Inspired Intelligence, Shanghai, 201210 China

## Abstract

**Background:**

Along with the development of precision medicine, individual heterogeneity is attracting more and more attentions in clinical research and application. Although the biomolecular reaction seems to be some various when different individuals suffer a same disease (e.g. virus infection), the final pathogen outcomes of individuals always can be mainly described by two categories in clinics, i.e. symptomatic and asymptomatic. Thus, it is still a great challenge to characterize the individual specific intrinsic regulatory convergence during dynamic gene regulation and expression. Except for individual heterogeneity, the sampling time also increase the expression diversity, so that, the capture of similar steady biological state is a key to characterize individual dynamic biological processes.

**Results:**

Assuming the similar biological functions (e.g. pathways) should be suitable to detect consistent functions rather than chaotic genes, we design and implement a new computational framework (ABP: Attractor analysis of Boolean network of Pathway). ABP aims to identify the dynamic phenotype associated pathways in a state-transition manner, using the network attractor to model and quantify the steady pathway states characterizing the final steady biological sate of individuals (e.g. normal or disease). By analyzing multiple temporal gene expression datasets of virus infections, ABP has shown its effectiveness on identifying key pathways associated with phenotype change; inferring the consensus functional cascade among key pathways; and grouping pathway activity states corresponding to disease states.

**Conclusions:**

Collectively, ABP can detect key pathways and infer their consensus functional cascade during dynamical process (e.g. virus infection), and can also categorize individuals with disease state well, which is helpful for disease classification and prediction.

## Introduction

The general gene-set or gene module can be used to find combinatory biomarkers or signatures to indicate particular phenotypic change of a biological system, e.g. the disease development or worsening [1–3]. However, such ab initio predictions usually have less interpretability in biological researches [4]. Different from these conventional methods, the pathway centered analysis can provide a new way to balance the discovery of interpretable biological functions and the detection of new pathway elements. Thus, these approaches could highlight the conditional importance of prior-known pathways [5] in a phenotype change/ transition [6], and also uncover its new candidate underlying functions [7].

Biological pathway consists of a set of interactive genes or other biomolecules, which is well-known to execute a series of functional cascades for particular cellular response/outcome [8]. Nowadays, there are many carefully curated pathways available to represent creditable functional compositions and interactions [9], which are expected to targetedly capture the permutation of established biological functions involved in the phenotype changes [10, 11].

Indeed, pathway centered models and methods have been widely applied in diverse biological and clinical studies. For instance, the well-known gene set enrichment analysis (GSEA) [12] can recognize dys-regulated pathway according to the measurement of status change of a pathway. Similarly, many pathway-level aggregation methods have been designed to investigate the biological signatures of different phenotypes on the pathway activity level [13]. PAGODA can reveal multiple overlapping aspects of transcriptional heterogeneity for coordinated variability amongst tested cells, by evaluating biological pathways as persistent cell-type specific features or transient processes [14]. The mendelian randomization-based pathway enrichment analysis (MRPEA), has developed a pathway association analysis method for correcting the genetic confounding effects of environmental exposures during the genetic studies of human complex diseases [15]. Pathifier is a principle curve based algorithm to infer pathway deregulation scores for each tumor sample on the basis of expression data by transforming gene-level information into pathway-level information [16]. A silico Pathway Activation Network Decomposition Analysis (iPANDA) is designed for robust biomarker identification from gene expression data, which estimates the pathway activation scores based on the degree of differential gene expression and pathway topology decomposition [17]. There are many further improvements on these pathway-centered computational approaches and their applications by machine learning ideas and technologies [18–20]. A network-based pathway-expanding approach is applied to take the topological structures of biological networks into account [21]; especially, the interaction between internal and external genes of the pathway and between pathways, can accurately and reliably identify significant pathways related to the corresponding disease [22]. And a multiple kernel learning has been proposed to feature selection by separately per data type and by pathway membership, and this maximizes the amount of information used to build effective prognostic prediction due to its usage of all available data [23].

Actually, there exists some pathway-related studies based on the time-course experiment and data, e.g. a pathway-based phylogenetic approach [24], a web-based software program Network Painter [25], and a new Wnt signaling time-fit model [26]. However, there is still urgent requirement on the computational characterization of interactive pathways and their phenotype-associated states, which should provide a new viewpoint of network of networks for a biological system [27], and should also supply new strategy to state prediction for the phenotype change of a biological system.

**Fig. 1:**
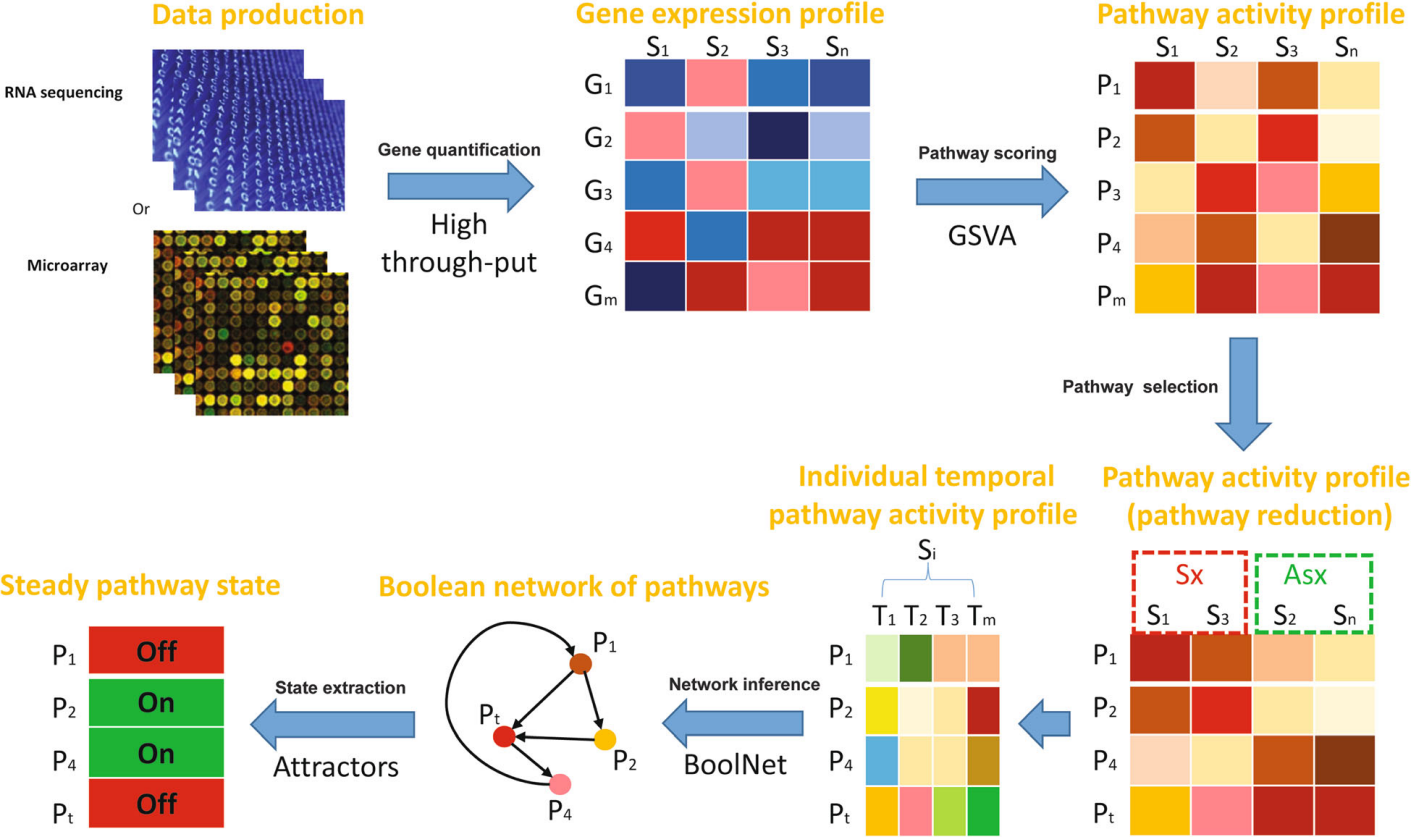
The framework ABP (Attractor analysis of Boolean network of Pathway) of pathway-centered dynamical network analysis. It includes several steps: transforming highthrough-put data into pathway score by GSEA; selecting dys-regulated pathways along time point; inferring network based on BoolNet; and finally computing attractors for each individual to describe the steady state of disease

Here, as shown in Fig. 1, we present a composite computational framework (ABP: Attractor analysis of Boolean network of Pathway) to investigate the dynamical biological process (e.g. state transition of biological systems). ABP reconstructs the Boolean network (BN) of pathways by the pathway activity profiles, and then applies the BN attractors to indicate the steady pathway states, which can quantitatively characterize the biological system status corresponding to different conditions or phenotypes (e.g. normal and disease). By wide evaluation on time-course gene expression datasets of virus infections, ABP has shown its efficiency and accuracy on identifying key phenotype-associated pathways subjected to virus infection; and rebuilding these key pathways’ consensus functional cascade during individual specific virus infection; and especially categorizing individuals with disease state well, which should be helpful for disease classification and prediction in personalized medicine.

## Methods

Our proposed ABP framework consists of a few main steps and parameters as illustrated in Fig. 1:

1. Normalization of gene expression data produced by high-throughput technologies, e.g. RNA sequencing or microarray;
2. Quantification of pathway activity according to pathway measuring approaches, i.e. GSVA using the expression data of each sample and gene-sets from KEGG database;
3. Selection of key phenotype-associated pathways through differential significance test (*P* < 0.05), where the number of finally selected pathways is dependent on the maximal number of significantly dys-regulated pathways at different time points;
4. Extraction of individual specific time-course pathway activity profile (i.e. every individual would have multiple samples collected at consecutive time points), thus, the organization of pathway activity profile is a cubic dataset, including key pathways, individuals, and time points; of note, the number of key pathways should be less than the number of time points, which is required by the follow-up BN construction;
5. Reconstruction of individual specific Boolean network based on corresponding time-course pathway activity profile, which is calculated by BoolNet method, using binarizeTimeSeries function with “scanStatistic” method, windowSize with intervals from 0.1 to 0.2, sign.level with intervals from 0.05 to 0.15; and applying reconstructNetwork function with “bestfit” method and default arguments.
6. Extraction of individual specific attractors corresponding to each Boolean network, which represent the individual specific steady pathway state so as to distinguish dissimilar phenotypes by unsupervised (i.e. HCL) or supervised (i.e. SVM with radial kernel) methods.

These key steps applied in above framework will be introduced in details in below.

### The calculation of pathway activity scores

Due to the reduced costs of high-throughput technologies, omics data is being produced under more complex experimental designs with multiple phenotypic and/or clinical samples. The Gene Set Variation Analysis (GSVA) [28] allows to assess the underlying pathway activity variation by transforming the gene by sample matrix into a gene set by sample matrix, which just provide the relative enrichment (scores) of pathways across the samples to support follow-up traditional analytical methods in a pathway focused manner, rather than the only qualitative enrichment with respect to a phenotype change. In this study, we apply GSVA to obtain the activity scores of KEGG pathways at multiple time points, which will be further used in following network and dynamic analysis on pathway level rather than gene level.

Notably, several approaches have proposed some pathway-level aggregation methods, but, it still remains unclear how they compare with one another due to limited evaluations. One recent benchmarking investigation has pointed that there is actually necessity for further improving pathway-level aggregation [29]. As known, there were remarkable outcome differences for different pathway tools even when the same data input. For example, the results of a tested approach were typically consistent across different datasets in cancer studies, yet different between methods [30].

Thus, any alternative kinds of activity score can also be used in this step if necessary in particular analysis routines, which would supply tunable analysis strategies dependent on researcher’s objective and experience.

### The rank of dys-regulated pathways

With an assumption of that the subjects / individuals with the same clinical symptoms should have the similar molecular / pathway reaction mechanism, the pathway activity may be various in individuals, but the final steady state of a network among pathways should be similar among the same symptom group or distinguishing between different symptom groups. Thus, we rank the differentially activated pathways between different phenotypic individual groups at each time point, which are dependent on the difference test of the activity scores by Wilcoxon rank sum test.

### The selection of key pathways during dynamical biological process

Then, according to the number of high-ranked dys-regulated pathways at each time point, we determine one critical time point, and the pathways significantly enriched (e.g. observed) at this time point are key pathways. These selected key pathways and their activities across consecutive time points can be regarded as the state indices which could trace the active pathway during a dynamical process, even in each subject. Noted, the final state or converged state (rather than currently observed state) of one pathway is characterized and quantified by the steady sate of this pathway in a subject, and this steady sate is modeled by network attractor and deduced from following Boolean network analysis.

### The re-construction of Boolean network among key pathways

To characterize the dynamical features of a biological system among above filtered key pathways, a network of pathways is constructed based on Boolean network model. At the starting point for a Boolean Network Model, *N* kinds of interacting nodes (i.e. pathways or meta-genes) are collected, the states of which are modeled as either “on” (active, or up-regulated) or “off” (inactive, or down-regulated). Then, at any given time, the system of such *N* key pathways is just in a system- or network-state. Along with a dynamical process, the system will change from one state to another depending on the interactions between these pathways. Thus, from any start state, the system is assumed to happen a confirmed sequence of state changes and ends up in a stable state (known as an attractor), and this sequence or trajectory of such system state change is defined as a Boolean process corresponding to discretized time-course data.

In practice, the Boolean network model is applied to convert the gene or pathway expression snapshots over a time course into a Boolean form by noting which genes or pathways are active or not. The dynamics of a Boolean network (BN) model (simply using the current state to determine the next state) can be described as follows:

$$ {\mathrm{s}}_{\mathrm{i}}\left(\mathrm{t}+1\right)=\left\{\begin{array}{c}1\ \sum \limits_{\mathrm{j}}{\mathrm{a}}_{\mathrm{j}\mathrm{i}}{\mathrm{s}}_{\mathrm{j}}\left(\mathrm{t}\right)>0\\ {}0\ \sum \limits_{\mathrm{j}}{\mathrm{a}}_{\mathrm{j}\mathrm{i}}{\mathrm{s}}_{\mathrm{j}}\left(\mathrm{t}\right)<0\\ {}{\mathrm{s}}_{\mathrm{i}}\left(\mathrm{t}\right)\ \sum \limits_{\mathrm{j}}{\mathrm{a}}_{\mathrm{j}\mathrm{i}}{\mathrm{s}}_{\mathrm{j}}\left(\mathrm{t}\right)=0\end{array}\right. $$

where, the s_i_(t) represents the state of some variable (e.g. a node in network) *i* at time point *t*; a_ji_ points the influence weight of another variable *j* for this variable *i;* thus, the state of variable *i* at time point *t + 1* would be determined by all other variables’ states at the time point ahead, i.e. at time point *t*.

In this study, the BoolNet package is used for the construction and evaluation of Boolean networks, which has been successfully applied in many biological and biomedical researches [31]. In particular, BoolNet is developed for the reconstruction and analysis of binary gene-regulatory networks: (i) the Network reconstruct function is used for inferring Boolean networks from temporal gene expression or pathway activity profiles by popular reconstruction algorithms; and (ii) the binarize Time Series function is used for binarizing the real-valued time series from these reconstruction algorithms. Noted, the tuning parameters used in BoolNet are searched by grid method and determined by the follow-up state clustering performance. Of course, some alternative Boolean network analysis methods are worthy of future benchmark investigations.

### The biological state clustering based on attractors of temporal network of key pathways

As assumed, the attractors represent a final steady state of a biological system, e.g. the success-infection or failure-infection states during virus infection. Obviously, these final states are expected to classify or even predict the particular phenotypes of individuals. Here, it is relevant to evaluate the indicative power of those state features rather than expression features, so that, the clustering of those states and expressions would be applicable and comparable, e.g. hierarchical clustering of pathway states and gene expressions respectively. However, in math terms, a state of an attractor is just a binary vector (attractor with simple structure) or a group of vectors (attractor with loop structure), so the conventional clustering method is not applied before we can supply the distance matrix among states. To generally measure the distance between two states (attractors), the canonical-correlation analysis (CCA) is used to infer the association information from cross-covariance matrices, which can indicate the association (distance) between two groups of vectors, i.e. two attractors.

Given two matrix X and Y, canonical-correlation analysis is to seek two vectors *a* and *b* to maximize the correlation between a ′ X and b ′ Y, i.e.

$$ \underset{\mathrm{a},\mathrm{b}}{\max}\uprho \left({\mathrm{a}}^{\prime}\mathrm{X},\mathrm{b}^{\prime}\mathrm{Y}\right)=\underset{\mathrm{a},\mathrm{b}}{\max}\frac{\mathrm{a}^{\prime }{\Sigma}_{\mathrm{XY}}\mathrm{b}}{\sqrt{\mathrm{a}^{\prime }{\Sigma}_{\mathrm{XX}}\mathrm{a}}\sqrt{\mathrm{b}^{\prime }{\Sigma}_{\mathrm{YY}}\mathrm{b}}} $$

where, Σ_XX_ = Cov(X, X), Σ_YY_ = Cov(Y, Y), Σ_XY_ = Cov(X, Y), and Cov(., .) is covariance matrix.

Then, the distance matrix converted from CCA matrix between multiple attractors corresponding to multiple individual Boolean networks, is used to build the hierarchical tree. Two clusters are divided, expecting one corresponds to Sx group and the other one corresponds to Asx group. And the clustering accuracy, defined as the application efficiency, is evaluated by the index Acc [32]. Given samples in Sx and Asx groups that are:

$$ {\left\{{\mathrm{S}}_{\mathrm{i}}\right\}}_{\mathrm{i}=1}^2 $$

where the state-based individuals clustering also provide two individual clusters:

$$ {\left\{{\mathrm{C}}_{\mathrm{j}}\right\}}_{\mathrm{j}=1}^2 $$

Then, the application efficiency of network reconstruction is calculated as:

$$ \mathrm{Acc}=\frac{\underset{\uptau \left(\left[1,2\right]\right)}{\min}\sum \limits_{\mathrm{j}=1}^2\mid {\mathrm{C}}_{\mathrm{j}}\sqcap {\mathrm{S}}_{\uptau \left(\mathrm{j}\right)}\mid }{\sum \limits_{\mathrm{i}=1}^2\mid {\mathrm{S}}_{\mathrm{i}}\mid } $$

Besides, the performance measurement of SVM adopts the AUC with random 5-fold cross-validation, which is used to evaluate the robustness of SVM features (i.e. pathway states as indicators).

## Results and discussion

### Instruction of datasets and experimental steps

To assess ABP, we collected and analyzed serially sampled gene expression data from a challenge study [27], noted as Rhinovirus UVA data. This data mainly contains 20 human volunteers (subjects) inoculated with live human rhinovirus (HRV), and each subject was serially sampled for a few days quantifying temporal whole blood gene expression by Affymetrix GeneChips technology, clinical symptom scores self-reported over 8–10 symptoms, and viral shedding from periodic nasopharyngeal titrations [33]. Each subject has different samples on 15 time points, one time point before viral Inoculum and the other 14 time points after inoculation. Each subject was designated as a symptomatic subject (Sx) or an asymptomatic subject (Asx) by a modified Jackson score based on these clinical symptoms self-reported [33]. The analyzed HRV UVA subjects were divided in to Sx group (10 individuals) and Asx group (6 individuals). Thus, actually, there were (10 + 6)*15 = 240 gene expression profiles used in this data study.

**Fig. 2:**
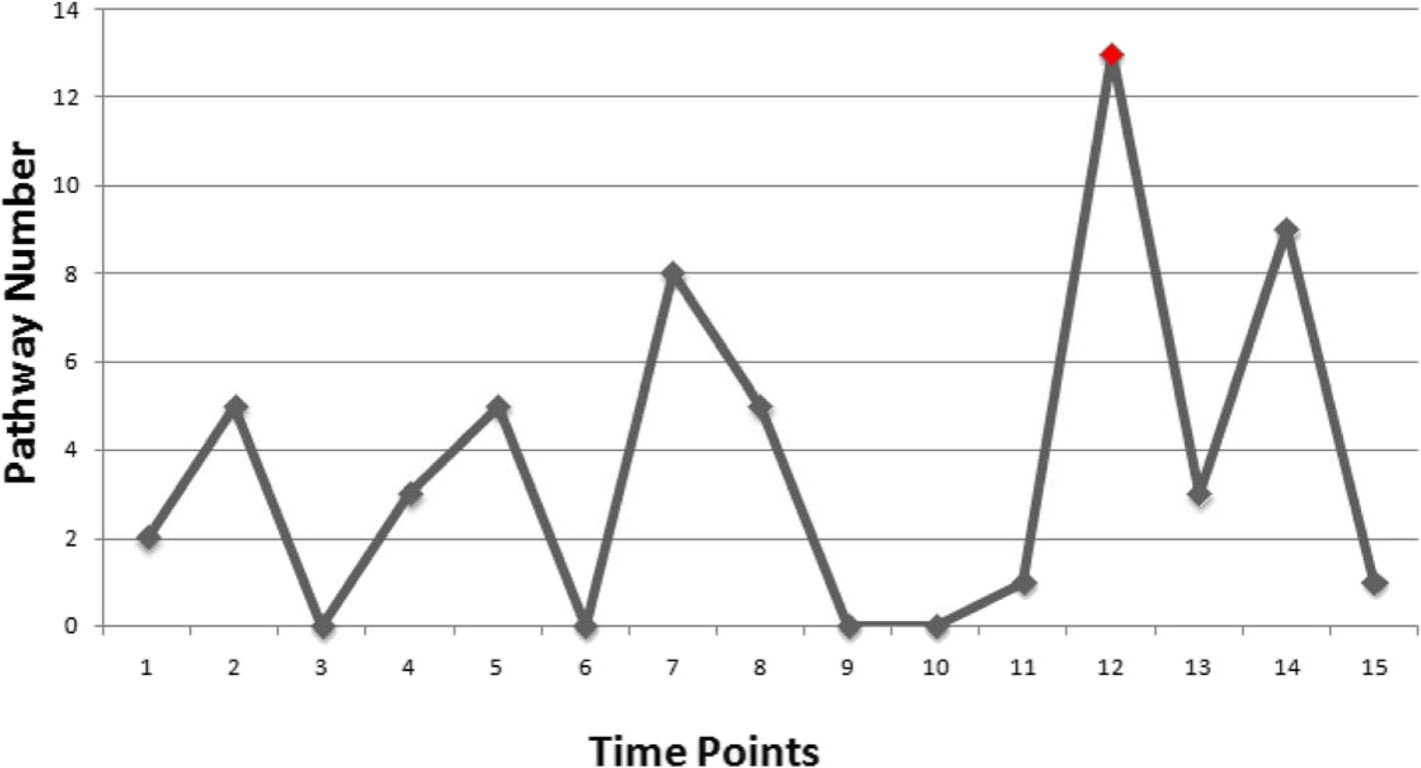
The number of dysfunctional pathways observed in different time points. The vertical axis of the histogram represents the number of dys-regulated pathways observed at particular time point; and the horizontal axis of the histogram represents the time points

Based on the pathway activities across multiple samples, we determined the critical time point and corresponding selected pathways. Seeing Fig. 2, the number of differentially activated pathways achieved maximum at 12_th_ time point (i.e. 48 h after inoculation), and 13 pathways significantly changed at this time point suggest a critical point between two groups of subjects. These selected pathways in Table 1 were applied as the activity indices aiming to trace the state change of pathways across consecutive time points in each subject, and the final state of pathways was determined by the steady sate of a Boolean network corresponding to each subject. Noted, at 14 _th_ time point, there were also nine dys-regulated pathways observed, only two of which were also observed at 12 _th_ time point (i.e. Fatty acid degradation and Glycine, serine and threonine metabolism), and the remains had less significance relevant to the studied disease. Thus, the selected 13 pathways at 12_th_ time point should be more pathogen informative in following analysis.

**Table 1:** The key pathways detected in HRV dataset

Metagene ID	Kegg ID	Pathway ID
Mgene 1	hsa00062	Fatty acid elongation
Mgene 2	hsa00260	Glycine, serine and threonine metabolism
Mgene 3	hsa00630	Glyoxylate and dicarboxylate metabolism
Mgene 4	hsa03320	PPAR signaling pathway
Mgene 5	hsa04115	p53 signaling pathway
Mgene 6	hsa04725	Cholinergic synapse
Mgene 7	hsa04750	Inflammatory mediator regulation of TRP channels
Mgene 8	hsa04920	Adipocytokine signaling pathway
Mgene 9	hsa04972	Pancreatic secretion
Mgene 10	hsa05143	African trypanosomiasis
Mgene 11	hsa05146	Amoebiasis
Mgene 12	hsa05160	Hepatitis C
Mgene 13	hsa05164	Influenza A

### Key pathways associated with individual phenotypes

Rhinovirus (RV) leads the majority of common colds, and also causes exacerbations in patients with asthma and chronic obstructive pulmonary disease. In the detected key pathways by ABP, many signaling pathways were efficiently detected and could be productive entry pathways for HRV. By examining the effects of tiotropium on RV infection and RV infection-induced airway inflammation, RV14 titres, RNA and cytokine concentrations would be reduced, along with the reduction of the expression of intercellular adhesion molecule and the number of cellular acidic endosomes modulating airway inflammation in rhinovirus infection [34]. Influenza virus or HRV infections of the upper airway can cause colds and the flu, and they can also activate exacerbations of lower airway diseases so as to induce chronic obstructive pulmonary disease and asthma, where a systems approach has identified the temporally changing patterns of host gene expression from these viruses [35], and a transmembrane protein RNASEK is needed for the replication of HRV, influenza A virus, and dengue virus [36]. In addition, a global study has also confirmed that RSV is an important respiratory pathogen in the elderly, and preventative measures such as vaccination could decrease severe respiratory illnesses and complications in the elderly [37]. Effective drugs and modification of pre-existing drugs or regimens would be improve to increase effectiveness of antiviral therapy, Pleconaril has some activity against enteroviruses and some efficacy against rhinoviruses in ongoing trials [38], e.g. it has been suggested that quercetin inhibits RV endocytosis and replication in airway epithelial cells at multiple stages of the RV life cycle [39].

In addition, a few our detected biological processes and functions are also associated with virus infection [40–42], such as HRV infection, as discovered in Table 1 (Noted, the metagene id in this table will be used to label the corresponding pathway in following figures for convenience). For example, the transient receptor potential (TRP) channel family are potential candidates for sensing physical and chemical stimuli, and TRP channels +may be novel therapeutic targets for controlling virus-induced cough [43]; Amoebiasis, the condition of harbouring the protozoan parasite Entamoebahistolytica, is a major health problem throughout the world [44], and parasite virulence can implicate multiple amoebic and host factors through complex host-parasite interactions [45]; and HCV infection is able to induce autophagy and downstream UPR molecules regulating key autophagic gene expression, which just can similarly promote human rhinovirus infection via the autophagic pathway [46, 47].

### Temporal module network charactering the dynamical process of individual phenotype appearance

**Fig. 3:**
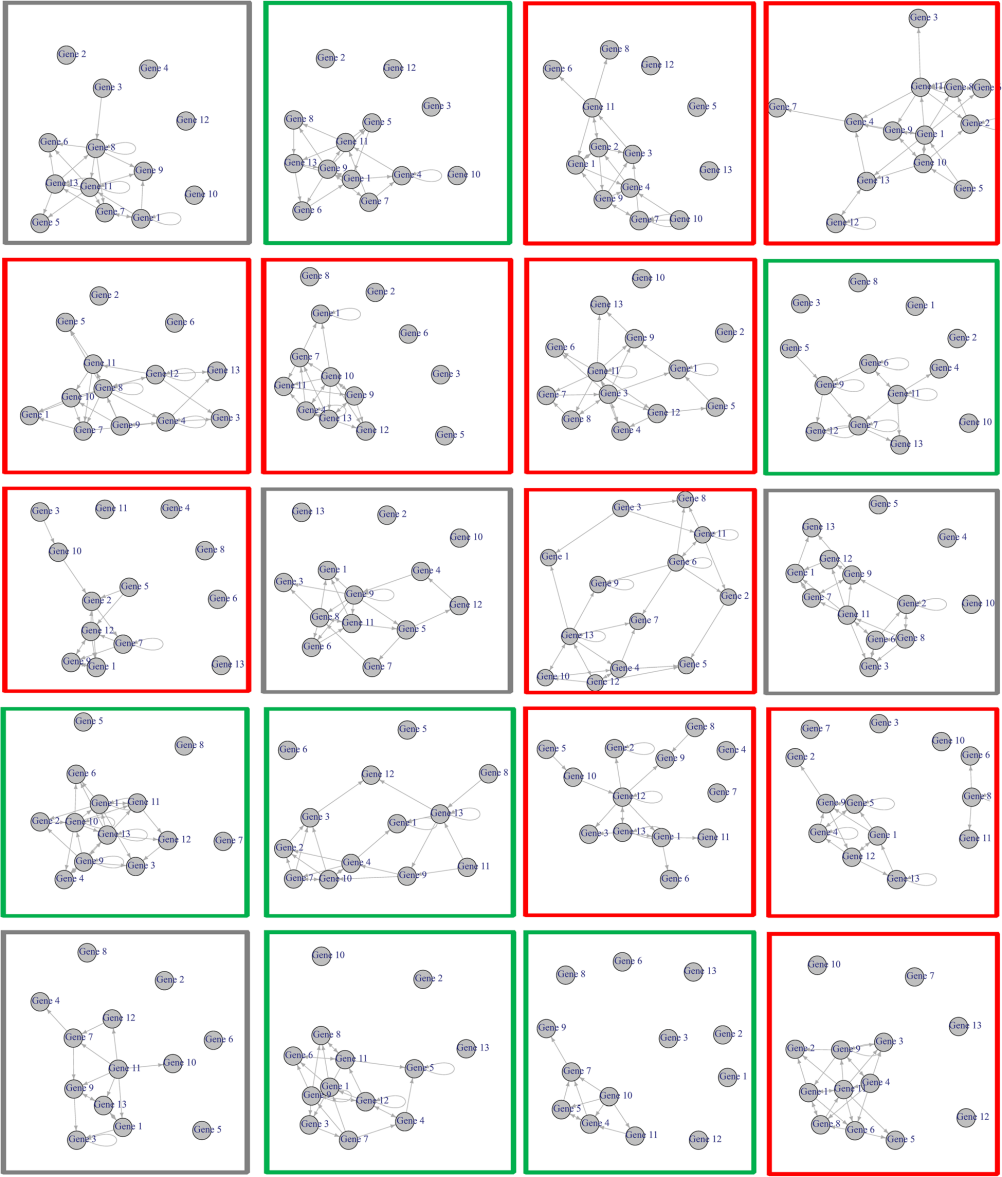
The topological structure of Boolean network corresponding to each subject. The Sx individual is labeled with red box; the Asx individual is labeled with green box; and the individuals labeled with grey box have not determinate clinical phenotype evidences in original report. Noted, each gene id is actually a metagene id which represents a particular pathway listed in Table 1

For each subject / individual, the topological structure of his / her Boolean network is displayed in Fig. 3. Obviously, these structures can’t classify the Sx and Asx groups directly, although there were some group specific edges observed in the networks. For instance, the regulation association between “Hepatitis C” and “Influenza A” pathways, has many observations in the Sx-individual specific networks (5 in 10) rather than in the Asx-individual specific networks (0 in 6), which is represented by the edge (meta-)Gene12 - > (meta-)Gene13; or the regulation association between “Amoebiasis” and “Glycine, serine and threonine metabolism” pathways, also has some observations in the Sx-individual specific networks (4 in 10) but none in the Asx-individual specific networks (0 in 6), which is represented by the edge (meta-)Gene11 - > (meta-)Gene2. Besides, there is a cross-reactivity between hepatitis C virus and Influenza A virus reported, and the host responses to an infectious agent can be influenced by such cross-reactive memory cells [48]. Thus, the “Hepatitis C” and “Influenza A” pathways, along with their involved genes / proteins, and even their interaction could all play important roles in HRV infection through a transferable manner.

### Attractor of module network indicating the pathways states corresponding to phenotypes

**Fig. 4:**
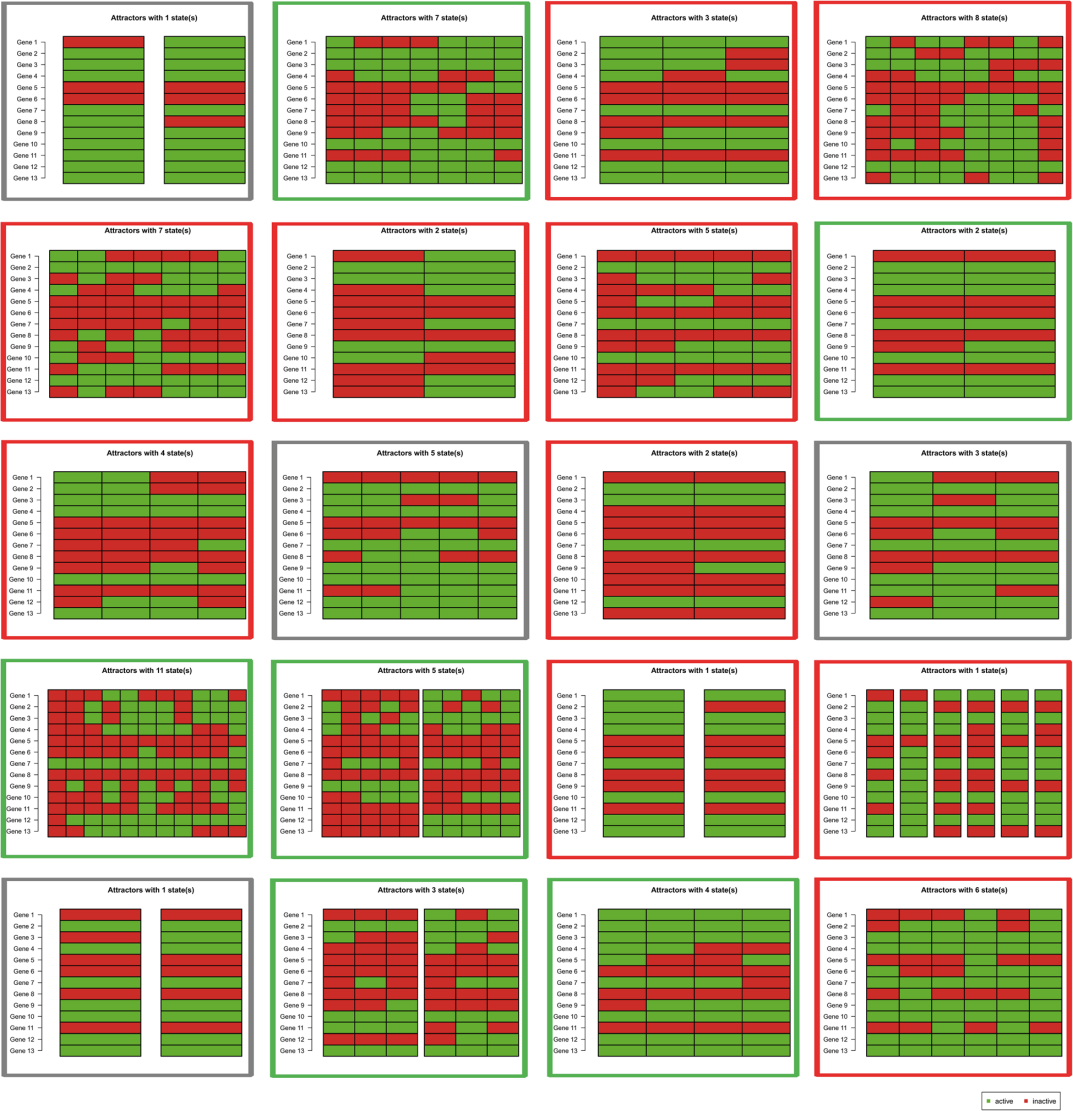
The attractor states of Boolean network corresponding to each subject. The Sx individual is labeled with red box; the Asx individual is labeled with green box; and the individuals labeled with grey box have not determinate clinical phenotype evidences in original report

**Fig. 5:**
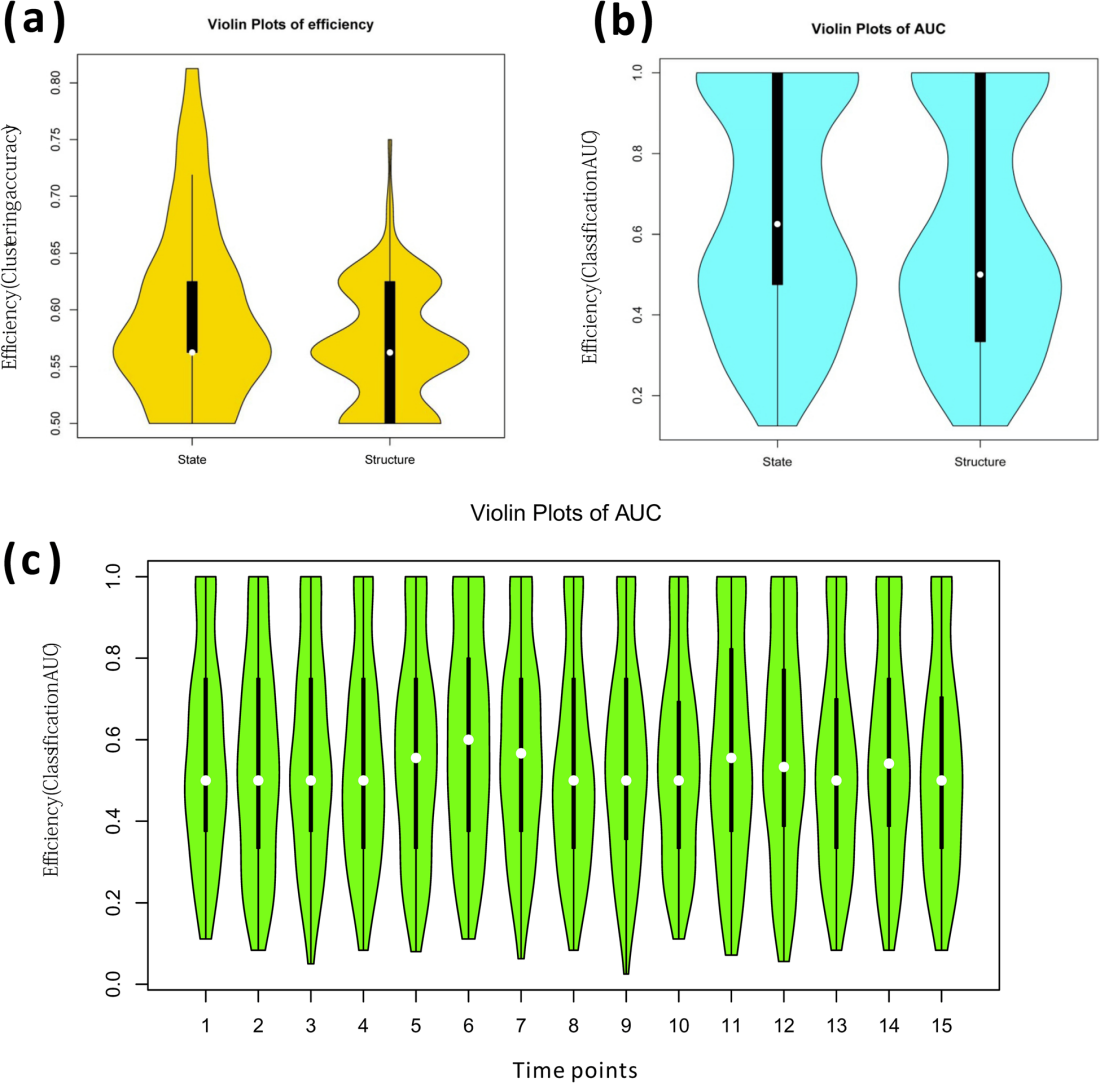
The efficiency for discriminating Sx and Asx individuals by the state and structure of Boolean network respectively, with statistic of Acc under different parameters. **a** The comparison between pathway activity based model and pathway network based model by Acc from clustering performance. **b** The comparison between pathway activity based model and pathway network based model by AUC from classification performance. **c** The robustness evaluation of pathway activity based model in a bootstrap manner

In addition to the aforementioned comparison and discussion about topological structures of individual specific BN, we further compared the individual specific final states of BN represented by the network attractors of networks. As illustrated in Fig. 4, there were several obvious common pathways observed in active states for Sx or Asx subjects, e.g. Hepatitis C and Influenza A, indicating the shared molecular mechanism suffering from virus infection. And most other pathways will be active or not for different subjects, but not distinguish for Sx and Asx groups separately. By contrast, integrating all pathways’ states, a simple clustering (i.e. HCL) can distinguish the Sx and Asx individuals with an accuracy larger than 80%, and would be more efficient than that based on topological structures in a parameter space, as displayed in Fig. 5a. Next, the AUC of SVM with 5-fold cross-validation had also been used to evaluate the robustness of our adopted network attractor based classification model. The results in Fig. 5b illustrated again the classification model trained from pathways’ states can achieve higher AUC than the classification model trained from pathways’ topological structures. In addition, in a bootstrap manner, by removing the samples at each time point, the attractor based model had been re-built and its performances kept well (i.e. with robustness) as shown in Fig. 5c. These results supported again the merit of this systems biology research, which provided discriminative systematical features (i.e. network features) to characterize the phenotype diversity, and also revealed the interpretable mechanisms underlying individual specific phenotypic changes. It should be noted that, although there are only 20 individuals in this data, the number of samples is actually larger than 200. And in the efficiency evaluation by hierarchical clustering or SVM, there are indeed 13 features used, which is less than the number of observations of model. Thus, the performance obtained here is not trivial. Of course, the evaluation on more individuals with multiple temporal samples should be further carried on in future work.

### Replicate discovery on independent dataset

**Fig. 6:**
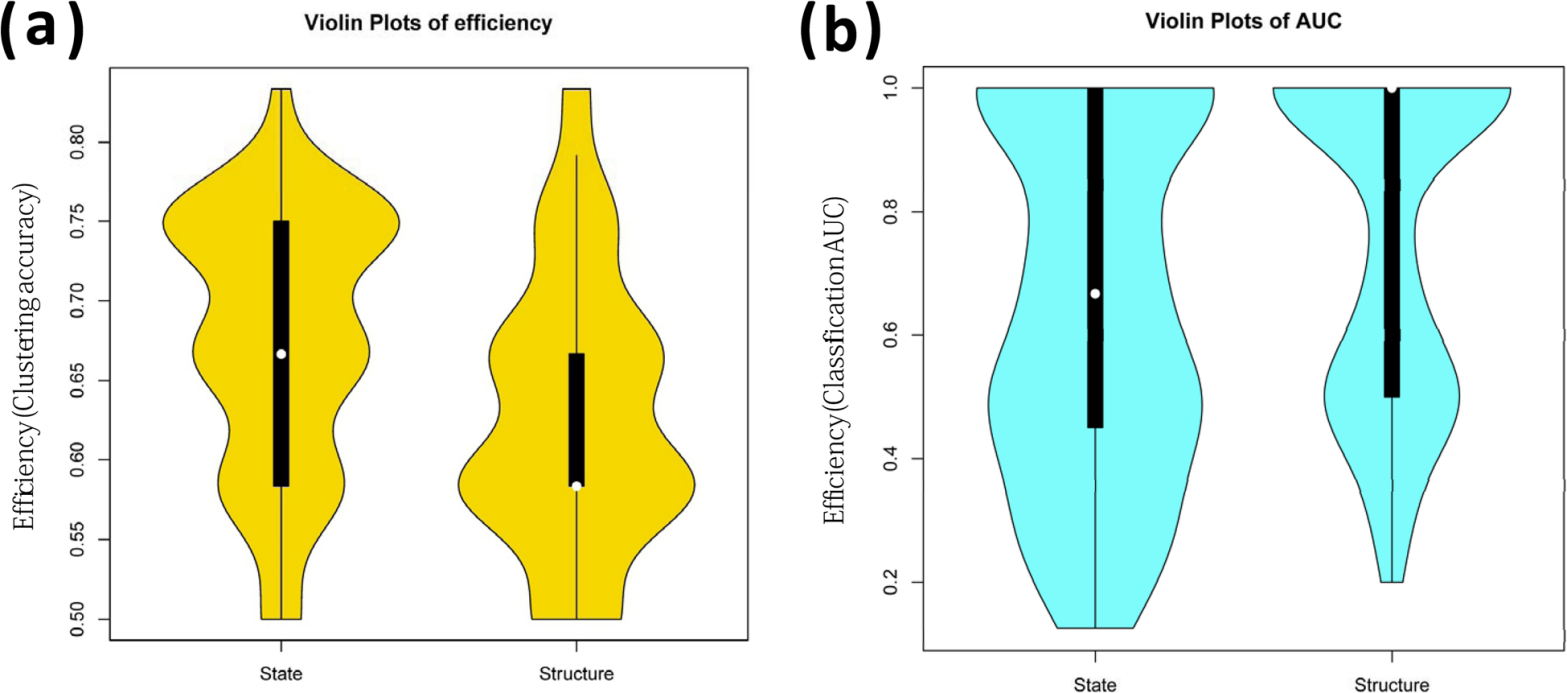
The efficiency for discriminating Sx and Asx individuals by the state and structure of Boolean network respectively on independent dataset, with statistic of Acc under different parameters. **a** The comparison between pathway activity based model and pathway network based model by Acc from clustering performance. **b** The comparison between pathway activity based model and pathway network based model by AUC from classification performance

To further validate the robustness of ABP, we used another gene expression data also inoculated with live human rhinovirus (HRV) [27], and this dataset was referred to Rhinovirus Duke data. This new dataset includes serially sampled gene expression data from 19 human volunteers (subjects). Each subject has different samples in 19 time points, one time point before the viral Inoculum and the remaining 18 time points behind inoculation. That means there are actually 19*19 = 361 expression profiles here. According to a modified Jackson score computed from the self-reported clinical symptoms, the analyzed HRV Duke subjects were also divided in to Sx group (9 individuals) and Asx group (3 individuals) and others [27]. We used the same key pathways with an assumption that the pathway reaction mechanism would be similar in the same disease, and more importantly this can also validate key pathways in an independent test. From the results on this dataset, we actually observed again that Boolean network model can mimic well a dynamical process that the biological system changes from one state to another. Especially, the steady state of the network attractors can distinguish Ax and Asx individuals efficiently (Fig. 6), which was also robust on parameters than that based on network structures. Notably, in the unsupervised evaluation as clustering in Fig. 6a, the steady state of the network attractors obviously outperformed the structure of network. Meanwhile, in the supervised evaluation as classification in Fig. 6b, the steady state of the network attractors tended to have satisfactory AUC performance (e.g. > 0.8) on larger parameter scopes than the structure of network, although the median performance of the structure of network seemed to be higher possibly caused by sample unbalance.

## Conclusions

In this study, we designed and implemented a computational framework ABP to investigate the dynamic of biological process for individual comparisons, especially on the network of pathways rather than genes. Based on the quantification of pathways activity (i.e. estimated activity score based on expression), the Boolean networks are reconstructed for each individual, so that the important network attractor can be obtained and applied to infer the final stable biological system state of individuals after particular phenotypic change (e.g. virus infection). Noted, some other pathway score like Pathifier has also been tested, however, the results show it is weak to obtain reasonable Boolean network, which would be caused by these scores being limited into a numeric region that would greatly affect the following network inference. Thus, the optimized combination of pathways activity estimation and Boolean network construction should be deserved to future study. Actually, both the structure and the attractor of the Boolean network can reveal indicative features (e.g. pathway associations or pathway activations) distinguishing Sx individuals or its sub-group from the Asx individuals. They would provide candidate disease identification or prediction approaches when there are enough individuals with temporal samples available [49].

Indeed, our proposed ABP has shown its efficiency and accuracy on identifying phenotype-associated pathways. These identified pathways could explain the phenotypic change in a state-transition manner, which is strongly supported by wide analysis and evaluation on multiple temporal gene expression datasets of HRV infections. A future direction is to further investigate how to build attractor-focused disease prediction model [50], and especially to release the model implementation as a web service for wide bioinformatics and biomedical study and application.

## Data Availability

The R codes are available at https://github.com/ztpub/Attractor-analysis-of-Boolean-network-of-Pathway.

## Abbreviations

ABP: Attractor analysis of Boolean network of Pathway
AUC: Area Under Curve
BN: Boolean network
CCA: Canonical-correlation analysis
GSEA: Gene set enrichment analysis
GSVA: Gene Set Variation Analysis
HCL: Hierarchical clustering
HCV: Hepatitis C virus
HRV: Human rhinovirus
iPANDA: A silico Pathway Activation Network Decomposition Analysis
KEGG: Kyoto Encyclopedia of Genes and Genomes
MRPEA: Mendelian randomization-based pathway enrichment analysis
PAGODA: Pathway and gene set over-dispersion analysis
RV: Rhinovirus
SVM: Support Vector Machine
